# The Association Between Facilitator Competent Adherence and Outcomes in Parenting Programs: a Systematic Review and SWiM Analysis

**DOI:** 10.1007/s11121-023-01515-3

**Published:** 2023-03-08

**Authors:** M. Martin, B. Steele, T. F. Spreckelsen, J. M. Lachman, F. Gardner, Y. Shenderovich

**Affiliations:** 1 Department of Social Policy and Intervention, University of Oxford, Oxford, UK; 2 School of Social and Political Sciences, University of Glasgow, Glasgow, UK; 3 Centre for Social Science Research, University of Cape Town, Cape Town, South Africa; 4 Wolfson Centre for Young People’s Mental Health, Cardiff, UK; 5 Evaluation, Complexity and Implementation in Public Health Improvement (DECIPHer), School of Social Sciences, Centre for the Development, Cardiff University, Cardiff, UK

**Keywords:** Parenting, Fidelity, Behavioral interventions, Systematic review, Violence prevention

## Abstract

There is increasing interest about the fidelity with which interventions are implemented because it is theorized that better implementation fidelity by facilitators is associated with better participant outcomes. However, in the parenting program literature, there is mixed evidence on the relationship between implementation fidelity and outcomes. This paper provides a synthesis of the evidence on the relationship between facilitator delivery and outcomes in the parenting program literature. Following PRISMA guidelines, this paper synthesizes the results of a systematic review of studies on parenting programs aiming to reduce violence against children and child behavior problems. Specifically, it examines associations between observational measures of facilitator competent adherence and parent and child outcomes. A meta-analysis was not feasible due to study heterogeneity. As a result, Synthesis Without Meta-Analysis guidelines were followed. Searches in electronic databases, reference searching, forward citation tracking, and expert input identified 9653 articles. After screening using pre-specified criteria, 18 articles were included. The review found that most studies (*n* = 13) reported a statistically significant positive relationship with at least one parent or child outcome. However, eight studies reported inconsistent findings across outcomes, and four studies found no association with outcomes. The results suggest that better facilitator competent adherence is generally associated with positive parent and child outcomes. However, this finding is weakened by the methodological heterogeneity of included studies and due to the wide variety of ways in which studies conceptualized competent adherence-outcome relationships.

**Supplementary Information:**

The online version contains supplementary material available at 10.1007/s11121-023-01515-3.

## Background

In recent years, there has been increasing interest in the fidelity with which evidence-informed interventions are implemented as it is theorized that better implementation fidelity — the extent to which an intervention is implemented as intended (Dane & Schneider, 1998) — is associated with better participant outcomes. Systematic review and meta-analytic evidence from a variety of fields now empirically supports that improving implementation fidelity is an important mechanism through which to enhance participant outcomes (Carroll et al., 2007). In the field of health promotion, a well-cited systematic review of over 500 studies reported evidence of a relationship between higher implementation fidelity and improved participant health and well-being at the study level (Durlak & DuPre, 2008). In the field of educational interventions, several systematic reviews found a positive relationship between fidelity and outcomes. A review of randomized trials found that higher levels of teacher fidelity were associated with improved student achievement outcomes (Hill & Erickson, 2019). Further, a review of the implementation of 29 school-based physical activity programs found program delivery by more highly competent teachers consistently predicted better student outcomes (Naylor et al., 2015), and a meta-analysis of 221 school-based child behavior programs found that implementation fidelity was a key contributor to positive changes in student behavior (Wilson et al., 2003).

Substantial evidence demonstrates that parenting programs are an effective way to support parents to acquire the knowledge and skills to enhance their children’s health and well-being and thereby improve child outcomes (e.g., Barlow & Coren, 2018; Barlow et al., 2006; Chen & Chan, 2016; Furlong et al., 2013; Knerr et al., 2013). However, the role of implementation fidelity and its relationship to intended outcomes in these programs is unclear. Few studies report on parenting intervention fidelity (Gardner et al., 2016), and among these, there is limited evidence on the relationship between implementation fidelity and outcomes (Rojas-Andrade & Bahamondes, 2019). One exception is a recently published systematic review of 24 studies on programs aiming to reduce child externalizing behaviors (Leitão et al., 2020). It reported on the role of several facilitator factors, including facilitator adherence, on program outcomes (Leitão et al., 2020). While this review concluded that facilitator delivery mattered for outcomes, it only included interventions specifically addressing child behavior problems and did not summarize the results of each study in detail. Another example is a meta-analysis of 156 studies on nine home visiting programs aiming to reduce child maltreatment which found that several implementation fidelity components, including facilitator adherence, were positively related to reductions in child maltreatment (Casillas et al., 2016).

Among the growing number of primary studies on the relationship between fidelity and outcomes, the evidence is inconsistent with some studies finding an association and others finding no association (Breitenstein et al., 2010; Cantu et al., 2010; Durlak, 1998; Fixsen et al., 2005; Forgatch et al., 2005; Olofsson et al., 2016). Some studies have found the relationship between fidelity and outcomes to be curvilinear wherein the highest levels of fidelity were detrimental to participant outcomes (e.g., Hogue et al., 2008). The literature may be inconsistent due to a variety of challenges connected with studying the relationship between fidelity and outcomes, including publication bias; the potential influence of confounding variables (Breitenstein et al., 2010); interaction effects with other aspects of implementation (Berkel et al., 2011); inaccurate measurement due to the use of tools which have not had their reliability and validity established (Breitenstein et al., 2010); insufficient power to examine the relationships due to small sample sizes; and little variation in fidelity or intervention outcomes, making analyses lack sensitivity to discern associations.

Given the widespread dissemination of parenting programs, the relationship between fidelity and outcomes should be clarified. Knowledge about the role that fidelity plays in outcomes would lead to an enhanced understanding of whether implementation is one of the key mechanisms via which parenting programs achieve their positive results for children and families. Such an understanding could then be used to inform future program delivery, particularly as programs are translated into community settings and taken to scale (Fixsen et al., 2005; Mowbray et al., 2003). As implementation fidelity is potentially an important factor in enhancing parenting program outcomes, there is a need to take stock of the existing literature on the relationship between fidelity and outcomes.

This paper synthesizes the research on the relationship between implementation fidelity and outcomes found via a systematic review of the existing literature on parenting programs aiming to (a) reduce child maltreatment; harsh or dysfunctional parenting; and/or child conduct problems and/or (b) improve positive child behavior management strategies; parent–child bonding/attachment and relationships; and/or early childhood development outcomes. The review focused on two aspects of implementation fidelity articulated in Proctor et al.’s taxonomy (2011) — adherence (strictness with which a facilitator implements the prescribed content) and competence (skill and style with which a facilitator delivers program components). Although distinct, these aspects were selected for this review as it is commonly thought that they should be assessed simultaneously (Forgatch et al., 2005; Breitenstein et al., 2010; Martin et al., 2021). Together, competence and adherence (or “competent adherence”) refers to the quality and strictness with which facilitators deliver an intervention as intended (Carroll et al., 2007; Forgatch et al., 2005). This review included studies that reported on competence and/or adherence.

This study is the first synthesis of the evidence on the relationship between observational measures of facilitator competent adherence and parenting program outcomes and summarizes the methods used to study the relationship. It specifically focuses on observational assessments of facilitator competent adherence — completed on facilitator program delivery based on their live or video-taped delivery — as these are considered most rigorous and provide a more detailed account of program delivery (Dusenbury et al., 2003; Eames et al., 2008). Further, the study provides critical insight into whether better fidelity is associated with improved family outcomes.

## Methods

This study builds on a systematic review conducted by Martin et al. (2021) that compiled the observational and non-observational measures of facilitator competent adherence found in the parenting program literature and synthesized the psychometric properties of the measures found. Using articles reporting on observational measures of facilitator competent adherence from the review, this paper synthesizes the evidence on the association, if any, between facilitator competent adherence and parent and/or child outcomes.

### Systematic Review

The full details of the methods used for the systematic review are described by Martin et al. (2021). In sum, a tested search strategy was implemented in 12 electronic bibliographic databases (see Online Resource 1). To find additional studies, the database searches were supplemented by reviewing articles included in a systematic review of parenting programs in low- and middle-income countries to ensure representation from these contexts (Gardner et al., forthcoming); conducting backward citation tracking; conducting forward citation tracking using Google Scholar; and seeking input from parenting program experts. The inclusion and exclusion criteria applied are summarized in Table 1. The review tested inter-rater reliability between two coders at the title/abstract, full-text, and data extraction stages. Percentage agreements ranged from 92.8 to 94.4% and were thus sufficiently high.

**Table 1:** Inclusion and exclusion criteria

**Inclusion criteria**	**Exclusion criteria**
Report on parenting programs aiming to (a) reduce child maltreatment; harsh or dysfunctional parenting; and/or child conduct problems and/or (b) improve positive child behavior management strategies; parent–child bonding/attachment and relationships; and/or early childhood development outcomes	Parenting programs with other aims or programs which (1) narrowly focus on specific child risks such as poisoning or accidents or on skills training for children’s specific medical conditions or physical disabilities (e.g., developmental disability) or (2) primarily deliver financial support (e.g., conditional case transfer programs) or other support to parents, but which do not aim to change parents’ knowledge or behavior concerning their child(ren)
Report on parenting programs wherein at least 50% of the program content is delivery to parents/caregivers	Parenting programs wherein children or others (not parents/caregivers) are the main focus of the intervention
Report on observational measures of facilitator competence and/or adherence	Reports solely on treatment alliance or working relationship Reports on facilitator competence and/or adherence without some reference to how it was measured or reports on a non-observational measure of facilitator competent adherence
Report on parenting programs wherein parents are 18 years or older and children are 17 years or younger	Reports on parenting programs for teenage parents (17 years and younger) and their children
Data surfacing from academic or grey publications including peer-reviewed articles, unpublished manuscripts, ongoing studies, and theses/dissertations	Data surfacing from books, newspapers, and magazines

**Fig. 1:**
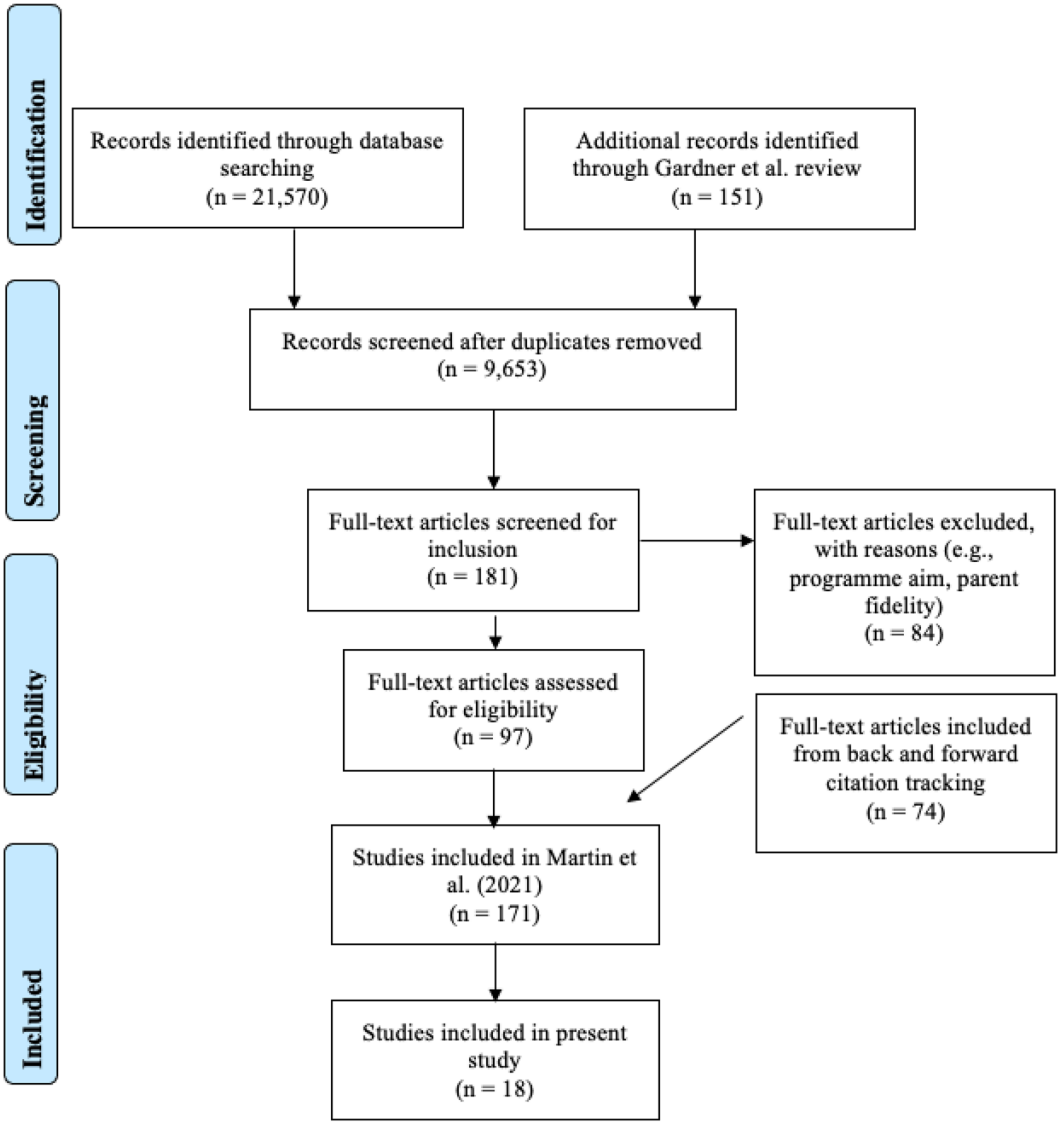
PRISMA flowchart of study screening and selection

The systematic review by Martin et al. found 9653 articles as of August 2021 (see Fig. 1). To be included in the review, articles must have been written in English; reported on observational measures of facilitator competent adherence; and used an observational, quasi-experimental, or experimental approach to analyze the association between facilitator competent adherence and family outcomes. Of the original articles, 18 articles reported on the relationship between observational measures of facilitator competent adherence and parent and child outcomes and were thereby included in the review.

#### Synthesis Without Meta-Analysis

Upon reviewing the 18 included studies, a meta-analysis was deemed methodologically unfeasible. As a result, a synthesis without meta-analysis was performed based on the Synthesis Without Meta-Analysis (SWiM) guidelines outlined by Campbell et al. (2020) (see Online Resource 2). These guidelines specify nine key categories of information that should be provided when a quantitative synthesis is not possible. The revised Meta-Analysis Reporting Standards was followed where possible (Appelbaum et al., 2018).

#### Reporting

Findings are reported following the PRISMA guidelines (Liberati et al., 2009) (see Online Resource 3). For studies which differentiated competent adherence-outcome relationships by subscale (e.g., adherence subscale score, competent subscale score, overall competent adherence score), overall competent adherence scores were extracted.

## Results

The 18 included studies are disparate in terms of the programs studied, parent and child outcomes considered, and methods used. This heterogeneity meant that a meta-analysis could not be conducted. Further, the small number of studies limited the feasibility of meta-analyzing subgroups. The results of the 18 studies are summarized in Table 2 and Online Resource 4. Using a modified version of the Thomson and Thomas (2013) effect direction plot visual display system, results are visualized using: a sideways arrow **(⇔)** indicating no statistically significant association between competent adherence and parent/child outcomes; an upwards arrow **(⇑)** indicating a positive, statistically significant association between stronger competent adherence and better parent/child outcomes; or a downwards arrow **(⇓)** indicating a negative, statistically significant association between stronger competent adherence and poorer parent/child outcomes.

**Table 2:** Summary of study results

**Paper**	**Program and country**	**Prevention classification**	**Fidelity measure**	**Mean fidelity (SD)**	**Measure timepoint**	**Findings**	**Summary**
(Cantu et al., 2010) or “S1”	Strengthening Families Program, US	Universal	Adherence only; assessed by researchers	81.00% (7.00%)	Immediately post-intervention	Adherence was not related to program outcomes	**Parenting: ⇔**
(Chiapa et al., 2015) or “S2” *data Smith et al. (2013)	Family Check-Up, US	Treatment	Competent adherence; assessed by a third party	61.10% (14.20%)	5.5–6.5 years post-registration	Decline in competent adherence associated with less behavior change (drift)	**Child behavior: ⇑**
(Eames et al., 2010) or “S3”	Incredible Years BASIC program, UK	Treatment	Competent adherence; assessed by a third party	Not reported	Immediately post-intervention	Facilitator competent adherence associated with better parenting skills	**Parenting: ⇑**
(Forgatch et al., 2005) or “S4”	Parent Management Training Oregon Model, US	Selected	Competent adherence; no assessor information	Not reported	12 months post-intervention	Higher competent adherence predicted improved parenting	**Parenting: ⇑**
(Forgatch & DeGarmo, 2011) or “S5”	Parent Management Training Oregon Model, Norway	Indicated	Competent adherence; assessed by trained non-participant observer	67.78% (15.67%)	9 months post-intervention	Higher competent adherence predicted improved parenting	**Parenting (path analysis): ⇑** **Parenting (correlations):⇔**
(Giannotta et al., 2019) or “S6”	Incredible Years, Sweden	Treatment	Competent adherence; assessed by independent raters	76.50% (14.40%)	Immediately post-intervention	Competent adherence was not associated with parent and child outcomes	**Parenting: ⇔** **Child behavior: ⇔**
(Hukkelberg & Ogden, 2013) or “S7”	Parent Management Training Oregon Model, Norway	Indicated	Competent adherence; assessed by researchers	73.12% (11.80%)	Timepoint 4 (not fully described)	Competent adherence predicted reductions in behavior issues (parent-report only); found alliance and competent adherence to be independent from each other; data only presented for timepoint three	**Child behavior (parent-report): ⇑** **Child behavior (teacher-report):⇔**
(Hogue et al., 2008) or “S8”	Multi-dimensional Family Therapy, US	Treatment	Adherence and competence measured separately; assessed by program supervisors	Parent interventions: Adherence: 67.71% Competence: 80.29% Family intervention: Adherence: 46.87% Competence: 74.57%	At 6-month follow-up	Better adherence predicted greater reductions in parent-reported externalizing but not youth-reported externalizing behavior Competence did not predict either internalizing or externalizing behavior as reported by parents and youth Some evidence of a curvilinear relationship for adherence-internalizing relationship, but not reported for externalizing despite this being a main outcome Curvilinear relationships between competence and outcomes are not explored Only report aggregate results not broken down by timepoint	**Adherence:** **Parent-reported externalizing (linear): ⇑** **Youth-reported externalizing (linear): ⇔** **Parent-reported internalizing (linear): ⇔** **Parent-internalizing (curvilinear): ⇑** **Competence:** **Parent-reported internalizing (linear): ⇔** **Parent- and youth-reported externalizing (linear): ⇔** **Externalizing curvilinear not reported**
(Maaskant et al., 2016) or “S9”	Parent Management Training Oregon Model, Netherlands	Universal	Competent adherence; assessed by program supervisors	79.80% (5.30%)	Both post-intervention and 4-month follow-up	Higher competent adherence associated with better improvements in some parenting dimensions but not others	**Post-test:** **Stress: ⇑** **Warmth: ⇔** **Responsiveness: ⇔** **Explaining: ⇑** **Autonomy:⇑** **Strictness: ⇔** **Discipline: ⇔** **Child behavior: ⇔** **Follow-up:** **Stress: ⇑** **Warmth: ⇔** **Responsiveness: ⇑** **Explaining: ⇑** **Autonomy: ⇑** **Strictness: ⇔** **Discipline: ⇔** **Child behavior: ⇔**
(Rendu, 2004) or “S10”	BASIC Parent-Training Program, UK	Treatment	Competent adherence; assessed by researchers	76.25%	5–7 months post-intervention	Some facilitator competent adherence dimensions associated	**Group facilitation and child behavior: ⇔ and ⇑** **Practicalities and child behavior: ⇔**
(Robbins et al., 2011) or “S11”	Brief Strategic Family Therapy, US	Treatment	Competent adherence; assessed by researchers	74.00%	12 months post-randomization	Competent adherence associated with better parent and child outcomes	**Family functioning: ⇑** **Drug use: ⇑** **Some outcomes not reported**
(Roggman et al., 2016) or “S12”	Early Head Start, US	Selected	Competent adherence; assessed by a third party	57.28% (8.00%)	Child reaches age 36 months (registration at 5 months)	Competent adherence associated with better parent and child outcomes	**Parenting: ⇑** **Child academics: ⇑**
(Satterfield, 2013) or “S13”	Functional Family Therapy, Ireland	Treatment	Competent adherence; assessed by program supervisors	66.00% (16.20%)	Final treatment	Competent adherence predicted behavior reductions from parent perspective but not teen perspective	**Youth reported behavior: ⇔** **Parenting reported behavior:⇑**
(Scott et al., 2008) or “S14”	Incredible Years, UK	Treatment	Competent adherence; assessed by researchers	68.00% (8.00%)	5–7 months post-intervention	Greater competent adherence predicted better behavior	**Child behavior: ⇑**
(Smith et al., 2013) or or “S15”	Family Check-Up, US	Treatment	Competent adherence; assessed by researchers	62.11% (14.22%)	One and 2 years post-intervention	Greater competent adherence not directly associated with better improvements in parenting and behavior	**Parenting: ⇔** **Child behavior: ⇔**
(Snider, 2019) or “S16”	Parent–Child Interaction Therapy, US	Universal and treatment	Competent adherence; assessed by researchers	Competence: 56.00% (32.00%) Adherence: 70.00% (25.00%)	Baseline, 3-month follow-up, 6-month follow-up, and 12-month follow up (model includes all points as growth)	Competent adherence not associated with child behavior or parenting; results for all timepoints not reported	**Child behavior: ⇔** **Parenting: ⇔**
(St. George et al., 2016) or “S17”	Familias Unidas, US	Selected	Competence and adherence measured separately; assessed by independent raters	51.43% (8.00%)	Baseline and 6-month follow-up	Higher competence associated with reductions in substance abuse but no relationship between adherence or competence and family functioning and no relationship between adherence and substance use	**Family functioning: ⇔ (but data not provided)** **Adherence and substance use: ⇔** **Competence and substance use: ⇑**
(Thijssen et al., 2017) or “S18”	Parent Management Training Oregon Model, Netherlands	Treatment	Competent adherence; assessed by independent raters	80.0%	Difference between scores at T1 (6 months), T2 (12 months), and T3 (18 months) with baseline	Associations were not significant, but sub-constructs of facilitator competent adherence were	**Child behavior: ⇔** **Parenting stress T1 and T2: ⇔** **Parenting stress and T3: ⇑**

Using a modified version of the Thomson and Thomas (2013) effect direction plot visual display system, results are visualized using: a sideways arrow **(⇔)** indicating no statistically significant association between competent adherence and parent/child outcomes; an upwards arrow **(⇑)** indicating a positive, statistically significant association between stronger competent adherence and better parent/child outcomes; or a downwards arrow **(⇓)** indicating a negative, statistically significant association between stronger competent adherence and poorer parent/child outcomes

### Programs and Outcomes

The 18 studies examined 11 different parenting programs delivered in high-income countries in Europe and the USA: Strengthening Families Program (*n* = 1; S1), Multi-Dimensional Family Therapy (*n* = 1; S8), Basic Parent Training Program (*n* = 1; S10), Brief Strategic Family Therapy (*n* = 1; S11), Early Head Start (*n* = 1; S12), Functional Family Therapy (*n* = 1; S13), Parent Child Interaction Therapy (*n* = 1; S16), Familias Unidas (*n* = 1; S17), Family Check-Up (*n* = 2; S2, S15), Incredible Years (*n* = 3; S3, S6; S14), and Parent Management Training Oregon Model (*n* = 5; S4, S5, S7, S9, S18). The studies reported an average of 38 facilitators and 159 families. The average level of fidelity reported in the studies was 69.46%. According to the Institute of Medicine (2009) classification system, two programs were universal prevention (S1, S9), two were indicated prevention (S5, S7), three were selected prevention (S4, S12, S17), 10 were treatments (S2, S3, S6, S8, S10, S11, S13, S14, S15, S18), and one was a combination of universal and treatment approaches (S16). Ten of the 18 studies included programs that targeted caregivers only, with the remaining eight targeting both caregivers and children (S1, S2, S8, S11, S13, S15–S17).

Studies assessed the association of competent adherence with five outcomes — child development (*n* = 1; S12), parenting stress (*n* = 2; S9, S18), family functioning (*n* = 2; S11, S17), parenting behaviors and skills (*n* = 9; S1, S3–S6, S9, S12, S15, S16), and child behavior (n = 13; S2, S6–S11, S13–S18). These outcomes were measured using numerous scales. For instance, in the 10 studies reporting on parenting behaviors and skills, the outcomes were measured using 10 different scales — the Intervention Targeted Parenting Attitude and Behavior Scale, the Dyadic Parent–Child Interaction Coding System, the Family and Peer Process Code, the Parenting Sense of Competence Scale, Parenting Behavior Questionnaire, the Home Observation Measure of the Environment, Relationships Process Code, Coders Impression Inventory, Alabama Parenting Questionnaire, and Caregiver Wish List. In the 13 studies reporting on child behavior, the outcomes were measured using eight different scales — the Child Behavior Checklist (which was used most often; CBCL), Eyberg Child Behavior Inventory (ECBI), Swanson Nolan and Pelham-IV Questionnaire (SNAP-IV), Strengths and Difficulties Questionnaire, Parent Account of Child Symptoms Interview, Parent Daily Report, and two different researcher-created self-reported drug use measures. The variability in the scales used to measure parent and child outcomes contributed to the methodological heterogeneity of the studies.

### Designs and Analysis Methods

The studies employed a wide range of methods. All studies examined facilitator delivery using data from the intervention arms of randomized trials. As expected, fidelity to outcome associations were observed as they occurred rather than experimentally manipulated. Associations between competent adherence and outcomes were analyzed using correlations (*n* = 1; S18), regression and one-way ANOVA (*n* = 1; S3), SEM/path analysis and correlation (*n* = 2; S5, S15), latent growth curve modeling (*n* = 3; S2, S8, S11), SEM/path analysis (*n* = 3; S7, S17, S4), and regression (*n* = 8; S1, S6, S9, S10, S12, S13, S14, S16). Competent adherence variables were modeled as categorical (*n* = 1, three categories: “no exposure,” “low exposure,” and “high exposure”; S3) or continuous (*n* = 17; all but S3) (Durlak & DuPre, 2008). Five studies conducted associations between fidelity captured at more than one timepoint, yet not all results were reported (S7, S8, S9, S16, S17).

#### Control Variables

Of the 18 studies, 17 reported controlling for potential confounding variables to estimate the relationship between competent adherence and outcomes (all but S18). The controls varied considerably with only two studies conducted by the same researcher reporting the same combination of variables. The control variables included therapeutic alliance, program site, baseline participant outcomes, facilitator characteristics, organizational characteristics, and participant characteristics (e.g., child age, child gender). For one study, control variables were only partially used as facilitator, organizational, family, and attendance variables were included in other parts of the structural equation model (S17). The studies did not discuss the rationale for selection of control variables.

#### Clustering and Multiple Comparison

Six of the 18 studies reported that they accounted for clustering of observations due to the nested design of delivering group-based parenting programs, often by facilitators working in pairs (S1, S4, S6, S8, S11, S14). Two of the five studies accounted for the same combination of clustering variables. The clustering variables accounted for included program level (*n* = 1; S1), within-couple dependence (*n* = 1; S4), family level (*n* = 1; S11), parenting group (*n* = 1; S6), and unspecified (*n* = 2, S8, S14). In one of the 18 studies, it was unclear whether clustering was used because multi-level modeling accounted for repeated measures and multiple respondents per family, but not for multiple families per facilitator (S9). Further, when associations with several outcomes are investigated, it is best practice to account for multiple comparisons. None of the studies reported accounting for multiple comparisons. 

### Associations of Competent Adherence with Outcomes

Most studies found that facilitator competent adherence was positively associated with one or more parent and/or child outcomes. Of the 18 studies, six found statistically significant positive associations (**⇑**) between competent adherence and all parent and child outcomes examined (S2–S4, S11, S12, S14). A further eight studies found mixed evidence (S5, S7–10, S13, S17, S18) wherein at least one outcome was positively associated with competent adherence and one outcome was not (**⇔**). Of these eight, all found that while some outcomes had a statistically significant positive association with competent adherence others had no significant association. None of the studies had a negative association with competent adherence. Of the 18 studies, four found no significant association between facilitator competent adherence and any of the outcomes studied (S1, S6, S15, S16). In conducting these analyses, most studies reported on the average level of fidelity achieved (all but S3 and S5).

#### Parenting Behaviors and Skills

Of the nine studies examining competent adherence and parenting behaviors and skills, three found a positive association (S3, S4, S12), two found mixed associations based on the two types of analyses performed (S5) or in the types of parenting behaviors measured (S9), and four found no associations (S1, S6, S15, S16).

#### Parenting Stress and Family Functioning

Of the two studies examining the relationship between competent adherence and parenting stress, one found a positive association (S9), and the other found mixed associations (S18). Similar findings were observed in the two studies examining competent adherence and family functioning wherein one found a positive association (S11) and one found no association (S17).

#### Child Behavior and Development

Of the 13 studies examining the relationship between competent adherence and child behavior outcomes, three found a positive association (S2, S11, S14); five found a mix of positive and no association based on who reported outcomes (e.g., parent- versus teacher-report) or based on varying dimensions of competent adherence examined (S8, S9, S10, S13, S17); and five found no associations (S6, S7, S15, S16, S18). Finally, in the one study examining child developmental outcomes, a positive association was observed between competent adherence and child academic attainment (S12).

#### Dimensions of Competent Adherence

Two studies examined competence and adherence separately and found differences in their association with outcomes (S8, S17). Utilizing linear models, Hogue et al. (2008; S8) found that adherence was related to greater reductions in child externalizing behavior while competence was not. Furthermore, neither adherence nor competence were associated with child internalizing behavior. In exploring curvilinear relationships, this study found some evidence of a curvilinear relationship between adherence and internalizing behavior where medium levels of adherence were positively associated with outcomes. However, the study did not report the results of an analysis of curvilinear relationships between adherence and externalizing behavior issues and did not report on competence and either internalizing or externalizing behavior issues. St. George et al. (2016; S17) found that competence was related to decreased substance use, but not related to improvements in family functioning and that adherence was not related to either reduced substance use or improved family functioning.

Four studies examined whether specific dimensions of competent adherence were associated with outcomes (S5, S10, S11, S18). For example, in one of two models tested, Rendu (2004; S10) found that one dimension of competent adherence — a group facilitation technique — was related to reductions in child behavior issues but another facilitation technique was not so related in either model tested. As another example, Robbins et al. (2011; S11) found that a facilitation approach called “joining” was associated with improved family functioning and reduced adolescent drug use, but three other facilitator approaches were not.

Several studies found differences in relationships between facilitator competent adherence and participant outcomes when reported using different measures (S7, S8, S13). To illustrate, in the paper by Hukkelberg and Ogden (2013; S7), competent adherence was associated with reductions in child behavior problems based on parent-reports, but not based on teacher-reports. As another example, the paper by Satterfield (2013; S13), competent adherence was associated with reductions in child behavior issues based on parent-reports, but no association was found based on youth-reports.

## Discussion of Clinical Implications

### Overall Findings

The synthesis considered 18 studies reporting on the relationship between observational measures of facilitator competent adherence and parent/child outcomes, with most interventions having a treatment focus. Studies focused variously on selective or indicated prevention, with most evaluating treatment programs. Treatment studies nevertheless have considerable implications for prevention, as treatment for child behavior problems is intended also to serve as prevention of their long-term adverse outcomes, including offending and poor mental health, education, and employment prospects. Of the 18 included studies, studies reported on child behavior, nine on parenting skills and behaviors, two on parenting stress, two on family functioning, and one on child development. The studies were highly heterogeneous in their design and analysis methods. Five studies conducted analyses on associations between fidelity captured at more than one timepoint.

Most studies found that facilitator delivery is associated with at least one parent or child outcome — eight of the 13 studies examining child behavior, five of the nine studies examining parenting skills and behaviors, both studies examining parenting stress, one of the two studies examining family functioning, and the one study examining child development. These findings generally suggest better competent adherence is associated with better parent and child outcomes. There was no discernible difference in associations between competent adherence and outcomes based on the aspects of competent adherence measured. Still, there was a sizeable number of studies with mixed findings where some outcomes are associated with competent adherence and others are not. Lack of detected associations between competent adherence and outcomes has several potential explanations, such as that fidelity does not matter for outcomes (low fidelity has no negative impact), that our efforts regarding fidelity are not worthwhile (high fidelity has no positive impact), issues due to poor measurement, and/or lack of statistical power to detect associations due to small sample sizes. Fidelity may also be indirectly associated with outcomes. Indirect associations were explored in several studies — such as Smith et al. (2013; S15) who found that although fidelity was not directly associated with parenting or child behavior, it was indirectly associated with some outcomes. Finally, other implementation variables, such as participant responsiveness or engagement during program sessions, participant attendance, and facilitator-participant working alliance, may mediate or moderate the relationship between fidelity and outcomes (Berkel et al., 2011; Carroll et al., 2007). For example, if the effect of fidelity on child outcomes is mediated by participant engagement, statistical models that adjust for this variable may reduce the observed relationship between fidelity and child outcomes. Further research on competent adherence-outcome relationships would benefit from a more systematic theoretical understanding of the variable relationships.

### Conceptualizing the Role of Facilitator Competent Adherence

The finding that better facilitator competent adherence is generally associated with better outcomes is limited by the diverse conceptualizations of the relationship between competent adherence and outcomes in the studies. This diversity is exemplified by the range of controls used in the models tested, including facilitator (e.g., therapeutic alliance), organizational (e.g., amount of coaching support provided to facilitators), and participant characteristics (e.g., child age, baseline outcomes), with only two studies using the same combination of controls. These differences reveal considerable variation in how researchers theorize about the potential mechanisms impacting, and dissensus on how they hypothesize, the relationship between facilitator competent adherence and outcomes. As few papers articulated a clear rationale or delineated a conceptual framework for their choice of controls, such as through directed acyclic graphs (DAGs) (Pearl et al., 2016), research in the field may be at risk of including unnecessary controls and overcontrol bias (incorporating inappropriate variables leading to spurious results or underpowered models) (Achen, 2005; Rohrer, 2018). As a result, future research would benefit from greater consideration and documentation of the theory underpinning the research — the mechanism(s) linking facilitator, organizational, and participant characteristics with competent adherence and its association with outcomes.

### Methodological Issues and Study Quality

There are limitations in the studies reviewed. In particular, issues concerning the robustness of the analyses, reliability and validity, and quality of reporting will be discussed using ROBINS-I (Risk of Bias In Non-randomized Studies of Interventions) (Sterne et al., 2016).

#### Robustness of Analyses

The sample size in the studies was generally small, with data collected from an average of 38 facilitators and 159 families. Further, none of the studies performed power calculations to determine the number of observations necessary to examine the relationship between competent adherence and outcomes or accounted for multiple comparisons. Studies differed considerably as to whether and which type of clustering was accounted for in the analyses. Clustering may occur at the parent group level (parents often receive an intervention in a group format) and the facilitator level (programs are often delivered by more than one facilitator and facilitators typically deliver a program to more than one parent) leading to non-independent observations. However, only six studies accounted for clustering. A wide range of statistical approaches (such as latent growth curve modeling, regression, and SEM/path analysis) were used, demonstrating variation in researcher thinking about how an analysis of competent adherence-outcome relationships should be examined. This variation was one factor which prevented a meta-analysis. If future studies report bivariate correlations and unstandardized regression coefficients, these studies will produce results that are easier to standardize and incorporate in meta-analyses.

#### Reliability and Validity

Little is known about the accuracy and reliability of observational measures of competent adherence meaning conclusions drawn from this synthesis should be made with caution (Breitenstein et al., 2010). Eleven of the 18 studies synthesized herein provide some information on the reliability and/or validity of the measures of competent adherence used — with ten of these reporting on inter-rater reliability, nine reporting on internal consistency, and five reporting on construct validity (Martin et al., 2021) (see Online Resource 4).

#### Reporting

In future research, study reporting could be more detailed. Five of 18 papers did not provide information on facilitator sample size. Additionally, several studies made claims about competent adherence-outcome relationships yet did not provide the numerical results. Other studies did not provide information about key outcomes mentioned in their methods.

### Strengths and Limitations

Although this paper makes an important contribution to implementation science as it relates to parenting programs, it has limitations. This review did not include studies reporting on non-observational measures of facilitator competent adherence (e.g., self-report measures). A synthesis of such measures would be worthwhile to conduct when greater study homogeneity permits meta-analyses. In addition, this review focused on parenting programs aiming to (a) reduce child maltreatment; harsh or dysfunctional parenting; and/or child conduct problems and/or (b) improve positive child behavior management strategies; parent–child bonding/attachment and relationships; and/or early childhood development outcomes. However, as studies reported on associations between facilitator competent adherence and several secondary outcomes (e.g., parenting stress), we synthesized information on these analyses as well. Thus, the associations reported herein between facilitator competent adherence and these secondary outcomes are likely not inclusive of all literature reporting on these outcomes. Further, this review was unable to explore selective reporting bias or publication bias. Aggregate associations and publication bias could not be explored as the substantial methodological heterogeneity prevented a meta-analysis from being conducted. While there are limitations, the review is the first to synthesize the data from studies examining the relationship between observationally measured facilitator competent adherence and parenting program participant outcomes to clarify the mixed evidence found in the literature.

### Suggestions for Future Research

Future studies, and the parenting intervention field at large, would benefit from investigations of competent adherence-outcome relationships that: clearly conceptualize the mechanisms hypothesized to influence competent adherence-outcome relationships; utilize larger sample sizes; account for clustering variables at the parent- and facilitator-level; incorporate carefully chosen control variables; follow best practices in open science, including pre-registration to minimize the risk of selective reporting; and report bivariate correlations and unstandardized regressions. As a result, this literature may benefit from reporting guidelines.

The field would also benefit from an examination of understudied and novel aspects of the relationship between competent adherence and family outcomes. For instance, there was some evidence from one study that the relationship between fidelity and outcomes was not linear and at high levels fidelity could be associated with worse program outcomes (Hogue et al., 2008). Exploration of curvilinear relationships would provide information on whether there is a tipping point at which further attention to fidelity is unnecessary or even unhelpful. Other topics to examine in future studies analyzing competent adherence-outcome relationships include examining the test–retest reliability of measures to see how competent adherence fluctuates over time as this review only identified two studies that examined associations between competent adherence and outcomes at more than one timepoint; determining how to weigh fidelity with adaptation; exploring whether competent adherence-outcome relationships are significant in the long-term; and testing whether fidelity is indirectly associated with parent/child outcomes or whether other implementation variables such as engagement or working alliance mediate the relationship between fidelity and outcomes*.* Further, it may be valuable to study competent adherence in different contexts, since all of studies found were nested within randomized trials, which may not always be fully representative of routine delivery contexts.

## Conclusion

This review aimed to provide clarity on the evidence regarding the role facilitator competent adherence plays in achieving parent and child outcomes. While this paper finds that the evidence is inconsistent, the synthesis also finds a general trend indicating that higher levels of facilitator competent adherence are related to improved parent and child outcomes. The latter finding is limited by a high number of the studies having found mixed evidence and no associations with outcomes as well as the diverse methodological approaches employed by the studies. The review highlights the need for further research on whether there is an association between facilitator competent adherence and outcomes and recommends how researchers and practitioners can advance the field.

### Supplementary Information

Below is the link to the electronic supplementary material.

Supplementary file1 (DOCX 24 KB)

Supplementary file2 (DOCX 26 KB)

Supplementary file3 (DOCX 31 KB)

Supplementary file4 (DOCX 55 KB)

## Data Availability

In line with TOP Guidelines, the plans for the systematic review and meta-analysis were pre-registered on PROSPERO (registration number: CRD42020167872). The paper analyzes a subset of the data used in a paper published in *Clinical Child and Family Psychology Review* (details provided in Online Resource 1). Two reporting guidelines were followed: Synthesis Without Meta-Analysis (Online Resource 2) and PRISMA (Online Resource 3). All study data used in the analyses have been made available at the Open Science Framework: https://osf.io/md2p7/?view_only=02b3b74f7ecb48668ee44b08e3c96007 and Online Resource 4.
