# Supplementary material for: The Association Between Facilitator Competent Adherence and Outcomes in Parenting Programs: a Systematic Review and SWiM Analysis
Source: Prev Sci. 2023 Mar 8;24(7):1314–26. doi: 10.1007/s11121-023-01515-3 (PMC10575799; doi:10.1007/s11121-023-01515-3)
Supplement: Supplementary file 1 — Supplementary file1 (DOCX 24 KB) [file 11121_2023_1515_MOESM1_ESM.docx]

The association between facilitator competent adherence and outcomes in parenting programs: A systematic review and SWiM analysis

Martin, M. ^1*^, Steele, B. ^1^, Spreckelsen, T. F. ^2^, Lachman, J. M.^1^, Gardner, F.^1^, & Shenderovich, Y. ^3, 4^

*Correspondence should be addressed to Mackenzie Martin, Department of Social Policy and Intervention, University of Oxford

Email: [Mackenzie.Martin@spi.ox.ac.uk](mailto:Mackenzie.Martin@spi.ox.ac.uk)

^1^Department of Social Policy and Intervention, University of Oxford, United Kingdom

^2^School of Social and Political Sciences, University of Glasgow, United Kingdom

^3^Wolfson Centre for Young People’s Mental Health

^4^ Centre for the Development and Evaluation of Complex Interventions for Public Health Improvement, School of Social Sciences, Cardiff University, United Kingdom

**Supplementary File 1 – Details of the Martin et al. 2021 review**

**Search String:**

(parent* OR caregiver* OR guardian* OR carer*.ab) AND (training OR program* OR intervention* OR treat- ment OR trial* or prevention.ab) AND (competen* OR qual- ity OR adheren* OR fidelity* OR integrity OR compliance. ab) AND (child* OR kid* OR adolesc* OR teen* OR youth* OR baby OR babies OR toddler* OR neonate* OR infant* OR newborn OR juvenile* OR minor* OR early child* OR ECD.ab) AND (facilitator* OR practitioner* OR therapist* OR clinician* OR teacher* OR worker* OR provider* OR leader* OR specialist* OR professional* OR coordinator* OR administrator* OR counsellor* OR counselor* OR implementer* OR coach* OR instructor* OR trainer* OR mentor* OR educator*.ab) AND (scale* OR subscale* OR tool* OR measure* OR instrument* OR report* OR index* OR checklist* OR test*.ab)

**Electronic Bibliographic Databases:**

Applied Social Sciences Index and Abstracts, Cochrane Database of Systematic Reviews, Cochrane Central Register of Controlled Trials (CENTRAL), EconLIT, PsycINFO, EBSCO combined search (CINAHL, ERIC, MEDLINE), Global Health, The International Bibliography of the Social Sciences (IBSS), Social Science Premium Collection, and ProQuest Dissertations and Theses

**Included Studies:**

Cantu, A. M., Hill, L. G., & Becker, L. G. (2010). Implementation quality of a family-focused preventive intervention in a community-based dissemination. *Journal of Children's Services, 5*(4), 18.

Chiapa, A., Smith, J. D., Kim, H., Dishion, T. J., Shaw, D. S., & Wilson, M. N. (2015). The trajectory of fidelity in a multiyear trial of the family check-up predicts change in child problem behavior. *Journal of Consulting and Clinical Psychology, 83*(5), 1006.

Eames, C., Daley, D., Hutchings, J., Whitaker, C. J., Bywater, T., Jones, K., & Hughes, J. C. (2010). The impact of group leaders’ behaviour on parents acquisition of key parenting skills during parent training. *Behaviour Research and Therapy, 48*(12), 1221-1226.

Forgatch, M. S., & DeGarmo, D. S. (2011). Sustaining fidelity following the nationwide PMTO™ implementation in Norway. *Prevention Science, 12*(3), 235-246.

Forgatch, M. S., Patterson, G. R., & DeGarmo, D. S. (2005). Evaluating fidelity: Predictive validity for a measure of competent adherence to the Oregon model of parent management training. *Behavior Therapy, 36*(1), 3-13.

Giannotta, F., Özdemir, M., & Stattin, H. (2019). The Implementation Integrity of Parenting Programs: Which Aspects Are Most Important? Child & Youth Care Forum,

Hogue, A., Henderson, C., Dauber, S., Barajas, P., Fried, A., & Liddle, H. (2008). Treatment adherence, competence, and outcome in individual and family therapy for adolescent behavior problems. *Journal of Consulting and Clinical Psychology, 76*(4), 544.

Hukkelberg, S. S., & Ogden, T. (2013). Working alliance and treatment fidelity as predictors of externalizing problem behaviors in parent management training. *Journal of Consulting and Clinical Psychology, 81*(6), 1010.

Maaskant, A. M., van Rooij, F. B., Overbeek, G. J., Oort, F. J., & Hermanns, J. M. (2016). Parent training in foster families with children with behavior problems: Follow-up results from a randomized controlled trial. *Children and Youth Services Review, 70*, 84-94.

Rendu, A. (2004). *Treatment adherence in a behaviourally based parenting program* University of London, University College London (United Kingdom)].

Robbins, M. S., Feaster, D. J., Horigian, V. E., Puccinelli, M. J., Henderson, C., & Szapocznik, J. (2011). Therapist adherence in brief strategic family therapy for adolescent drug abusers. *Journal of Consulting and Clinical Psychology, 79*(1), 43.

Roggman, L. A., Cook, G. A., Innocenti, M. S., Jump Norman, V., Boyce, L. K., Christiansen, K., & Peterson, C. A. (2016). Home visit quality variations in two early head start programs in relation to parenting and child vocabulary outcomes. *Infant Mental Health Journal, 37*(3), 193-207.

Satterfield, L. W. (2013). The Effectiveness of Functional Family Therapy in an Irish Context: An Examination of International Implementation.

Scott, S., Carby, A., & Rendu, A. (2008). Impact of therapists’ skill on effectiveness of parenting groups for child antisocial behavior. *Institute of Psychiatry, Kings College London*.

Smith, J. D., Dishion, T. J., Shaw, D. S., & Wilson, M. N. (2013). Indirect effects of fidelity to the family check-up on changes in parenting and early childhood problem behaviors. *Journal of Consulting and Clinical Psychology, 81*(6), 962.

Snider, M. D. (2019). Examining the Impact of Treatment Fidelity on Client Outcomes in a Statewide Implementation of Parent-Child Interaction Therapy. *Western Virginia University.*

St. George, S. M., Huang, S., Vidot, D. C., Smith, J. D., Brown, C. H., & Prado, G. (2016). Factors associated with the implementation of the Familias Unidas intervention in a type 3 translational trial. *Translational Behavioral Medicine, 6*(1), 105-114.

Thijssen, J., Albrecht, G., Muris, P., & de Ruiter, C. (2017). Treatment Fidelity during therapist initial training is related to subsequent effectiveness of parent management training—Oregon model. *Journal of Child and Family Studies, 26*(7), 1991-1999.
