# Supplementary material for: The Association Between Facilitator Competent Adherence and Outcomes in Parenting Programs: a Systematic Review and SWiM Analysis
Source: Prev Sci. 2023 Mar 8;24(7):1314–26. doi: 10.1007/s11121-023-01515-3 (PMC10575799; doi:10.1007/s11121-023-01515-3)
Supplement: Supplementary file 4 — Supplementary file4 (DOCX 55 KB) [file 11121_2023_1515_MOESM4_ESM.docx]

The association between facilitator competent adherence and outcomes in parenting programs: A systematic review and SWiM analysis

Martin, M. ^1*^, Steele, B. ^1^, Spreckelsen, T. F. ^2^, Lachman, J. M.^1^, Gardner, F.^1^, & Shenderovich, Y. ^3, 4^

*Correspondence should be addressed to Mackenzie Martin, Department of Social Policy and Intervention, University of Oxford

Email: [Mackenzie.Martin@spi.ox.ac.uk](mailto:Mackenzie.Martin@spi.ox.ac.uk)

^1^Department of Social Policy and Intervention, University of Oxford, United Kingdom

^2^School of Social and Political Sciences, University of Glasgow, United Kingdom

^3^Wolfson Centre for Young People’s Mental Health

^4^ Centre for the Development and Evaluation of Complex Interventions for Public Health Improvement, School of Social Sciences, Cardiff University, United Kingdom

| **Table S1**  *Summary of Study Data* | | | | | | | | | | | |
| --- | --- | --- | --- | --- | --- | --- | --- | --- | --- | --- | --- |
| **Paper** | **Program** | **Fidelity Domain Measured** | **Method** | **Sample Size** | **Associated Outcomes** | **Accounted for Clustering** | **Control Variables** | **Adjusted for Multiple Comparisons** | **Results** | **Findings** | **Summary** |
| (Cantu et al., 2010) or S1 | Strengthening Families Program in the United States | Composite measure of adherence | Regressions (unadjusted and multi-level); continuous approach | Facilitator = 47  Parents = 96 | Parenting skills and behaviours (parent-reported; Intervention Targeted Parenting Attitude and Behaviour Scale) | Yes (program level) | Yes (in multi-level model including participant characteristics, pre-test scores, program characteristic, facilitator characteristics) | No | Parenting skills and behaviours: B= 2.45, SE= 2.92, t=0.84, p=0.45 | Adherence was not related to program outcomes | **Parenting: ⇔** |
| (Chiapa et al., 2015) or S2  *data from Smith, 2013 | Family Check-Up in the United States | Competent Adherence (composite) | Latent growth curve modelling; continuous approach | Facilitator = 79  Families = 79 | Child behaviour (parent- and teacher-reported; Child Behaviour Checklist) | No | Yes (income, site, caregiver depression, therapist transfer, child behaviour problems at baseline) | No | Fidelity latent growth curve slope to child behaviour with covariates not included: *b*= -0.69, B= -0.95, posterior SD = 0.333, <-0.01  With covariates included: β = -0.95, p = 0.003, 95% CI (-2.11, -0.22) | Decline in competent adherence associated with less behaviour change (drift) | **Child behaviour: ⇑** |
| (Eames et al., 2010) or S3 | Incredible Years BASIC program in the United Kingdom | Two dimensions of competent adherence (facilitator praise and reflective statements) | Regression and one-way ANOVAs; categorical approach | Facilitator = Not reported  Families = 104 | Parental praise and reflexive statements (observer-reported; Dyadic Parent-Child Interaction Coding System) | No | Yes (baseline DPICs) | No | Facilitator reflective and parent reflective: B=0.20, p <0.05, R^2^=0.04  Facilitator praise and parent praise: B=0.35, p < 0.01, R^2^=0.12  ANOVA praise: (F[2, 150]=10.90, p < 0.01)  ANOVA reflective: (F[2,150]=4.81, p < 0.01) | Facilitator competent adherence associated with better parenting skills | **Parenting: ⇑** |
| (Forgatch et al., 2005) or S4 | Parent Management Training Oregon Model in the United States | Competent adherence (composite) | Multivariate SEM/path analysis; continuous approach | Facilitator = 4  Families = 20 | Parenting skills and behaviours (observer-reported; Family and Peer Process Code) | Yes (within-couple dependence) | Yes (baseline status, child age, child gender, encouragement sessions, discipline sessions) | No | Change in maternal parenting: B=0.51, p <0.05, R^2^=0.53  Change in step-father parenting: B=0.49, p < 0.05, R^2^=0.66 | Higher competent adherence predicted improved parenting | **Parenting: ⇑** |
| (Forgatch & DeGarmo, 2011) or S5 | Parent Management Training Oregon Model in Norway | Competent adherence (composite) | Bivariate correlations and SEM/path analysis; continuous approach | Facilitator = 35  Families = 242 | Parenting skills and behaviours (observer-reported; Family and Peer Process Code) | No | Yes (pre- and post- treatment, child age, child gender, encourage sessions, discipline sessions) | No | Parenting skills and behaviours: B= 0.17(0.23) with p<0.05 and R^2^=0.40(0.43) with multi-level parameters adjusted for clustering in brackets  FIMP encouragement and change in mother’s parenting: 0.05, p>0.05  FIMP discipline and change in mother’s parenting: 0.07, p>0.05  FIMP encouragement and change in father’s parenting: 0.07, p>0.05  FIMP discipline and change in father’s parenting: 0.10, p>0.05 | Higher competent adherence predicted improved parenting | **Parenting (path analysis): ⇑**  **Parenting (correlations):⇔** |
| (Giannotta et al., 2019) or S6 | Incredible Years in Sweden | Competent Adherence | Multi-level linear regression | Facilitator = 111  Parents = 535  Children = 749 | Parenting skills and behaviours (parent-reported; Parenting Sense of Competence Scale) and child behaviour (parent-reported; Eyberg Child Behaviour Inventory and Swanson Nolan and Pelham-IV) | Yes (maximum likelihood robust multi-level modelling to take parenting and groups into account) | Yes (child age, parent age, parent involvement, parent attendance) | No | Parent outcomes:  (1) angry outbursts = 0.01(0.05) with 0.00(0.01) residual, (2) harsh parenting = 0.02(0.13) with 0.00(0.02) residual, (3) attempted understanding = -0.01(.01) with 0.00(0.01) residual, (4) praise = -0.04(0.18) with .00(0.03) residual, (5) reward = -.08(0.11) with 0.01(0.13) residual, (6) competence = -0.02(0.03) with .00(.01) residual  Child outcomes: (1) ECBI = -0.01(0.02) with 0.01(0.02) residual, (2) ECBI problem = 0.01(0.01) with 0.01(0.01) residual, (3) inattention = -0.01(0.02) with 0.00(0.01) residual, (4) hyperactivity = -0.01(0.02) with 0.00(0.01) residual, (5) oppositional defiance = 0.02(0.02) with 0.00(0.01) residual | Competent adherence was not associated with parent and child outcomes | **Parenting: ⇔**  **Child behaviour: ⇔** |
| (Hukkelberg & Ogden, 2013) or S7 | Parent Management Training Oregon Model in Norway | Competent adherence | SEM/path analysis | Facilitator = 134  Families = 331 | Child behaviour (teacher-reports on Child Behaviour Checklist and parent-reports on Parent Daily Report) | No | Yes | No | Parent report: B= 0.18, p < 0.01, R^2^=0.49  Teacher report: B= -0.02, p > 0.32, R^2^=0.41, p > 0.05 | Competent adherence predicted reductions in behaviour issues (parent-report only); found alliance and competent adherence to be independent from each other | **Child Behaviour (parent-report): ⇑**  **Child Behaviour (teacher-report):⇔** |
| (Hogue et al., 2008) or S8 | Multi-dimensional Family Therapy in the United States | Competent and adherence measured separately | Latent growth curve modelling with pseudo z-tests; continuous approach | Facilitator = 5  Families = 36 | Child behaviour (parent- and youth-reported; revised  Child Behaviour Checklist) | Yes (accounted for nesting) | Yes (therapeutic alliance, outcomes, treatment condition and interaction terms) | No | Main Effects:  Parent-reported externalizing and adherence mean slope = -2.07, SE=0.87, pseudo-z = -2/36, p <0.05, 95% CI: -2.94, -1/18, d=0.37  Parent-reported internalizing and adherence mean slope: 0.27, SE=0.84, p>0.05  Parent-reported externalizing and competence mean slope: 0.53, SE=0.55, p>0.05  Parent-reported internalizing and competence mean slope: -0.44, SE=0.59, p>0.05  Youth-reported externalizing and adherence mean slope: -0.32, SE=0.63, p>0.05  Youth-reported externalizing and competence mean slope: -0.09, SE=0.54, p>0.05  Curvilinear Relationships:  Internalizing and adherence mean slope = -1.50, pseudo-z = 2.46, p <0.05, 95% CI: -2.11, 0.89, d=0.40 | Better adherence predicted greater reductions in parent-reported externalizing but not youth-reported externalizing behaviour  Competence did not predict either internalizing or externalizing behaviour as reported by parents and youth  Some evidence of a curvilinear relationship for adherence-internalizing relationship, but not reported for externalizing despite this being a main outcome  Curvilinear relationships between competence and outcomes are not explored | **Adherence:**  **Parent-reported externalizing (linear): ⇑**  **Youth-reported externalizing (linear): ⇔**  **Parent-reported internalizing (linear): ⇔**  **Parent-internalizing (curvilinear): ⇑**  **Competence:**  **Parent-reported internalizing (linear): ⇔**  **Parent- and youth-reported externalizing (linear): ⇔**  **Externalizing curvilinear not reported** |
| (Maaskant et al., 2016) or S9 | Parent Management Training Oregon Model in the Netherlands | Competent adherence | Multi-level regression; continuous approach | Facilitator = Not reported  Families = 86 | Parenting stress (parent-reported; Parenting Stress Index); parenting behaviours (parent-reported; Parenting Behaviour Questionnaire); and child behaviour (parent-reported; Child Behaviour Checklist) | Unclear – likely partially (multi-level modeling accounted for repeated measures and multiple respondents per family but not for multiple families per therapist/facilitator; number of facilitators not clear) | Yes (baseline outcome levels) | No | Baseline to Post-test:  Parenting stress: β=0.27, SE= 0.07, p < 0.01 at baseline and β=0.28, SE=0.09, p < 0.03 at follow-up  Parenting warmth: β= -0.02, SE=0.09, p <0.86 at baseline and β=0.15, SE=0.09, p < 0.17 at follow-up  Parenting responsiveness: β= 0.13, SE=0.12, p <0.31 at baseline and β=0.23, SE=0.09, p <0.05 at follow-up  Parent explaining: β=0.25, SE=0.09, p <0.03 at baseline and β=0.26, SE=0.11, p < 0.05 at follow-up  Parenting autonomy: β=0.34, SE=0.14, p <0.04 at baseline and β=0.32, SE=0.11, p < 0.03 at follow-up  Parent strictness: β= 0.01, SE=0.09, p < 0.95 at baseline and β=0.12, SE=0.11, p < 0.36 at follow-up  Parent discipline: β=0.17, SE=0.13, p < 0.31 at baseline and β=0.04, SE=0.11, p <0.69 at follow-up  Child behaviour: β=0.22, SE=0.10, p < 0.07 at baseline and β=0.21, SE=0.13, p <0.17  Baseline to Follow-Up:  Parent stress: B=0.28, SE=0.09, p<0.03  Parent warmth: B=0.15, SE=0.09, p<0.17  Parent responsiveness: B=0.23, SE=0.09, p<0.05  Parent autonomy: B=0.32, SE=0.11, p<0.05  Parent strictness: B=0.12, SE=0.11, p<0.69  Parent discipline: B=0.04, SE=0.11, p<0.69  Child behaviour: B=0.21, SE=0.13, p<0.17 | Higher competent adherence associated with better improvements in some parenting dimensions but not others | **Post-Test:**  **Stress: ⇑**  **Warmth: ⇔**  **Responsiveness: ⇔**  **Explaining: ⇑**  **Autonomy:⇑**  **Strictness: ⇔**  **Discipline: ⇔**  **Child Behaviour: ⇔**  **Follow-Up:**  **Stress: ⇑**  **Warmth: ⇔**  **Responsiveness: ⇑**  **Explaining: ⇑**  **Autonomy: ⇑**  **Strictness: ⇔**  **Discipline: ⇔**  **Child Behaviour: ⇔** |
| (Rendu, 2004) or S10 | BASIC Parent-Training Program in the United Kingdom | Competent adherence (measured in terms of ‘group facilitation’ and ‘practicalities’) | Linear regression; continuous approach | Facilitator = 13  Families = 84 | Child behaviour (parent-reported; Parents Accounts of Child Symptoms Interview) | No | Yes (model 1: pre-treatment child behaviour and model 2: pre-treatment child behaviour, child age, hyperactivity) | No | Group facilitation model 1: B=-0.19, SE= 0.08, Beta= -0.24, p = 0.01  Group facilitation model 2: B=-0.14, SE=0.08, Beta=.17, p = .09  Practicalities: B=-0.08, SE= 0.10, Beta= -0.09, p= 0.40  Practicalities model 2: B= -0.07, SE= 0.10, Beta= 0.07, p=0.45 | Some facilitator competent adherence dimensions associated | **Group facilitation and child behaviour: ⇔ and ⇑**  **Practicalities and child behaviour: ⇔** |
| (Robbins et al., 2011) or S11 | Brief Strategy Family Therapy in the United States | Competent adherence (broken down into four behaviours) | Latent growth curve modelling; continuous approach | Facilitator = 5  Families = 480 | Family functioning (parent- and youth-reported; Family Environmental Scale) and child behaviour (adolescent drug use; self-report measure) | Yes (nesting at family-level) | Yes (baseline outcomes) | No | One of the four behaviours was related to outcomes (“joining”)  Family functioning: b = 0.053, SE=0.019, p <0.005, standardized coefficient gamma=0.203  Adolescent drug use: b = .121, SE = .051, p < .018  The results of the other three behaviours are not reported | Some aspects of competent adherence associated with outcomes | **Family functioning: ⇑**  **Drug use: ⇑**  **Some outcomes not reported** |
| (Roggman et al., 2016) or S12 | Early Head Start in the United States | Competent adherence | Regression; continuous approach | Facilitator = Not reported  Families = 71 | Parenting behaviour and skills (observer-reported; Home Observation Measure of the Environment) and child development (Peabody Picture Vocabulary Test-III) | No | Yes (intervention site) | No | Parenting behaviour and skills: *B*=1.22, SE=0.62, β=0.52, p <0.001  Child vocabulary: *B*=6.88, SE=3.16, β=0.33 p < 0.05 | Competent adherence associated with better parent and child outcomes | **Parenting: ⇑**  **Child academics: ⇑** |
| (Satterfield, 2013) or S13 | Functional Family Therapy in Ireland | Competent adherence | Regression; continuous approach | Facilitator = 9  Families = 60 | Child behaviour (parent- and youth-reports; Strengths and Difficulties Questionnaire) | No | Yes (pre-test adolescent functioning) | No | Youth-report: B=-.410, p <0.642  Parent-report B=-2.87, p <.000 | Competent adherence predicted behaviour reductions from parent perspective but not teen perspective | **Youth reported behaviour: ⇔**  **Parenting reported behaviour:⇑** |
| (Scott et al., 2008) or S14 | Incredible Years in the United Kingdom | Competent adherence | Regression; continuous approach | Facilitator = 13  Families = 73 | Child behaviour (parent-reported; Parent Account of Child Symptoms Interview) | Yes (accounted for nesting using multi-level modelling) | Yes (child age, attendance, pre-treatment scores) | No | B=-0.25, SE=.09, p<0.05  A one-point increase in skill was associated with a 0.58 SD increase in child behaviour | Greater competent adherence predicted better behaviour | **Child behavior: ⇑** |
| (Smith et al., 2013) or S15 | Family Check-Up in the United States | Competent adherence | SEM/path analysis and correlations; continuous approach | Facilitator = Not reported  Families = 79 | Parent skills and behaviours (observer-reported; HOME; Relationships Process Code; Coders Impression Inventory) and child behaviour (parent-reported; Child Behaviour Checklist) | No | Yes (baseline parenting behaviour and skills, baseline child problem behaviour) | No | Correlation between competent adherence and parent behaviour and skills at age 2: r=0.14, not significant  Correlation between competent adherence and parent behaviour and skills at age 3: r=0.05, not significant  Correlation between competent adherence and child behaviour at age 2: r=0.14, not significant  Correlation between competent adherence and child behaviour age 3: r=0.09, not significant  Path analysis of competent adherence to parent behaviour and skills at age 3: *B*=-0.05, posterior SD=0.05, β=-0.10, 95% CI[-0.328, 0.091], p>0.01  Indirect effects (competent adherence -> engagement -> positive behaviour support -> problem behaviour: *B*=-0.24, posterior SD=0.19, 95% CI=-.664-3.019, p>0.05 | Greater competent adherence not directly but indirectly associated with better improvements in parenting and behaviour | **Parenting: ⇔**  **Child behaviour: ⇔** |
| (Snider, 2019) or S16 | Parent-Child Interaction Therapy in the United States | Competent adherence | Hierarchical linear modelling; continuous approach | Facilitator = 17  Families = 32 | Parenting behaviour and skills (parent-reported; Alabama Parenting Questionnaire) and child behaviour (parent-reported; Eyberg Child Behaviour Inventory) | No | Yes (early attribution but had no impact in model) | No | Fixed effects  ECBI Intensity  Adherence    b=6.70, SE= 3.28, p=.069  Competence b= -3.97, SE= 2.55, p= .150    ECBI Problem  Adherence b=2.07, SE= 0.95, p=.054  Competence b=-1.03, SE= 0.71, p= .179    APQ-9 Problem  Adherence b=-0.13, SE= 0.09, p=.187  Competence b= -0.05, SE= 0.07, p= .525 | Competent adherence not associated with child behaviour or parenting | **Child behaviour: ⇔**  **Parenting: ⇔** |
| (St. George et al., 2016) or S17 | Family Unidas in the United States | Competence and adherence measured separately | SEM/path analysis; continuous approach | Facilitator = Not reported  Families = 367 | Child behaviour (youth-reported; self-reported substance abuse) and family functioning (latent construct created from items from the Parent Relationship with Peer Group Scale, Parenting Practices Scale, and Family Relations Scale; parent-reported) | No | Partly (facilitator variables, organizational variables, family demographics, attendance, pre-test family functioning, pre-test adolescent substance use included in other parts of the structural equation model) | No | Adherence and family functioning: not associated and do not provide results  Adherence and substance use: b=−0.28, p< 0.50  Competence and family functioning: not associated and do not provide results  Competence and substance use: b=-.999, p < 0.05, 95% CI [=1.682, -0.315] | Higher competence associated with reductions in substance abuse but no relationship between adherence or competence and family functioning and no relationship between adherence and substance use | **Family functioning: ⇔ (but data not provided)**  **Adherence and substance use: ⇔**  **Competence and substance use: ⇑** |
| (Thijssen et al., 2017) or S18 | Parent Management Training Oregon Model in the Netherlands | Competent adherence | Pearson correlation; continuous approach | Facilitator = 25  Families = 86 | Child behaviour (parent-reported; Child Behaviour Checklist and Parent Daily Report), parenting stress (parent-reported; Nijmeegse Ouderlijke Stress Index and Symptom Checklist Revised), and parenting skills and behaviours (parent-reported; Caregiver Wish List) | No | No | No | Child behaviour (CBCL): r= -0.18 (T1), r= -0.05 (T2), r=-0.26 (T3)  Child behaviour (PDR): r=-0.02 (T1), r=0.09 (T2), r=0.03 (T3)  Parent stress (NOSI): r= -0.22 (T1), r=-0.13 (T2), r=-0.32 (p<0.05)  Parent Stress SCL-R: r= -0.12 (T1), r=-0.13 (T2), r=-0.21 (T3)  Parenting practices (CWL): r=0.22 (T1), r=0.05 (T2), r=0.21(T3) | Associations were not significant but sub-constructs of facilitator competent adherence were; association between facilitator competent adherence and parenting stress was significant at one time point | **Child behaviour: ⇔**  **Parent stress T1 and T2: ⇔**  **Parent stress T3: ⇑** |

| **Table S2**  *Location, gender, and ethnic make-up of study participants* | | | | |
| --- | --- | --- | --- | --- |
| **Paper** | **Program** | **Targeted** | **Female Participants** | **Ethnicity** |
| (Cantu et al., 2010) | Strengthening Families Program in the United States | Caregivers and children | Caregivers: 59.00%  Children: 47.00% | 68.00% European American, 11.00% Latino/a, 11.00% American Indigenous, 2.00% African American |
| (Chiapa et al., 2015)  *data from Smith, 2013 | Family Check-Up in the United States | Caregivers and children | Caregivers: 100.00%  Children: 49.00% | Not reported |
| (Eames et al., 2010) | Incredible Years BASIC program in the United Kingdom | Caregivers only | Not reported | Not reported |
| (Forgatch et al., 2005) | Parent Management Training Oregon Model in the United States | Caregivers only | Caregivers: 50.00%  Children: 25.00% | 88.2% European American, 1.8% Latino/a, 0.00% Indigenous American, 0.00% African American, 10.00% multiracial or other |
| (Forgatch & DeGarmo, 2011) | Parent Management Training Oregon Model in Norway | Caregivers only | Not reported | Not reported |
| (Giannotta et al., 2019) | Incredible Years in Sweden | Caregivers only | Caregivers: 85.00%  Children: not reported | Not reported |
| (Hukkelberg & Ogden, 2013) | Parent Management Training Oregon Model in Norway | Caregivers only | Caregivers: not reported  Children: 26.00% | Not reported |
| (Hogue et al., 2008) | Multi-dimensional Family Therapy in the United States | Caregivers and children | Caregivers: not reported  Children: 19.00% | 70.00% African American, 20% European American, 10% Hispanic American |
| (Maaskant et al., 2016) | Parent Management Training Oregon Model in the Netherlands | Caregivers only | Caregivers: not reported  Children: 54.00% | Not reported |
| (Rendu, 2004) | BASIC Parent-Training Program in the United Kingdom | Caregivers only | Caregivers: not reported  Children: 27.40% | Not reported |
| (Robbins et al., 2011) | Brief Strategy Family Therapy in the United States | Caregivers and children | Caregivers: not reported  Children: 21.46% | 44.38% Hispanic American, 30.83% European American, 22.90% African American |
| (Roggman et al., 2016) | Early Head Start in the United States | Caregivers only | Caregivers: 100.0.%  Children: 58.00% | 84.00% European American |
| (Satterfield, 2013) | Functional Family Therapy in Ireland | Caregivers and Children | Caregivers: not reported  Children: 35.00% | 93.30% White Irish, 1.70% White Romanian, 1.70% White Zimbabwean, 1.70% biracial Irish |
| (Scott et al., 2008) | Incredible Years in the United Kingdom | Caregivers only | Not reported | 20% ethnic minority |
| (Smith et al., 2013) | Family Check-Up in the United States | Caregivers and children | Caregivers: not reported  Children: 49.00% | 51.00% European American, 30.00% African American, 12.00% biracial, 7.00% Hispanic American, 1.00% Indigenous American |
| (Snider, 2019) | Parent-Child Interaction Therapy in the United States | Caregivers and children | Caregivers: 90.00%  Children: 25.00% | 84.00% European American |
| (St. George et al., 2016) | Family Unidas in the United States | Caregivers and children | Caregivers: not reported  Children: 48.00% | At least one caregiver per family identified as Hispanic American |
| (Thijssen et al., 2017) | Parent Management Training Oregon Model in the Netherlands | Caregivers only | Not reported | Not reported |

| **Table S3**  *Psychometric Evidence of Measures in Included Studies* | |
| --- | --- |
| **Paper** | **Psychometric Evidence**  **(see Martin et al., 2021)** |
| (Cantu et al., 2010) | No |
| (Chiapa et al., 2015) | Yes |
| (Eames et al., 2010) | No |
| (Forgatch et al., 2005) | Yes |
| (Forgatch & DeGarmo, 2011) | Yes |
| (Giannotta et al., 2019) | Yes |
| (Hukkelberg & Ogden, 2013) | Yes |
| (Hogue et al., 2008) | No |
| (Maaskant et al., 2016) | No |
| (Rendu, 2004) | Yes |
| (Robbins et al., 2011) | No |
| (Roggman et al., 2016) | Yes |
| (Satterfield, 2013) | No |
| (Scott et al., 2008) | Yes |
| (Smith et al., 2013) | Yes |
| (Snider, 2019) | Yes |
| (St. George et al., 2016) | Yes |
